# Inhibition of *Flavobacterium psychrophilum* biofilm formation using a biofilm of the antagonist *Pseudomonas fluorescen*s FF48

**DOI:** 10.1186/2193-1801-2-176

**Published:** 2013-04-22

**Authors:** Mery De la Fuente, José M Vidal, Claudio D Miranda, Gerardo González, Homero Urrutia

**Affiliations:** Biofilms and Environmental Microbiology Laboratory, Centro de Biotecnología, Universidad de Concepción, Concepción, Chile; Aquatic Pathobiology Laboratory, Departamento de Acuicultura, Universidad Católica del Norte, Coquimbo, Chile; Centro de Estudios Avanzados en Zonas Áridas (CEAZA), Coquimbo, Chile; Antibiotics Laboratory, Departamento de Microbiología, Universidad de Concepción, Concepción, Chile

**Keywords:** *Flavobacterium psychrophilum*, Biocontrol, Biofilm, Bacterial antagonism, Aquaculture

## Abstract

The most important bacterial pathology currently occurring in Chilean freshwater salmon farming is the cold-water disease produced by the psychrotrophic bacteria *Flavobacterium psychrophilum*. The main aim of this study was to characterize the inhibitory activity of an antagonist strain on the formation of biofilms of a *F*. *psychrophilum* strain. The antagonistic strain *Pseudomonas fluorescens* FF48 was isolated from the sediment beneath the salmon cages of a freshwater Chilean salmon farm and was identified by using the 16S rRNA gene sequence analysis. The production of siderophores, mainly during the stationary phase of growth of the antagonist strain was demonstrated using the Chrome Azurol S method and through *F*. *psychrophilum* inhibition under iron saturation conditions. Subsequently, the effect of the antagonist supernatant on the formation of *F*. *psychrophilum* biofilm was tested using the crystal violet staining method observing an inhibition of the growth of *F*. *psychrophilum*, but no effect was observed when iron saturation concentrations were used. Furthermore, when the antagonist strain was previously deposited on the support, it completely inhibited the formation of *F*. *psychrophilum* biofilms, but when both bacteria were inoculated simultaneously no inhibitory effect was detected. In conclusion, it was demonstrated that FF48 strain is able to inhibit the formation of *F*. *psychrophilum* biofilms *in vitro* probably mediated by the siderophore production, suggesting its potential use as a biocontrol biofilm in freshwater fish rearing systems to prevent the persistence of biofilms of the fish pathogenic species *F*. *psychrophilum*.

## Background

*Flavobacterium psychrophilum* is a widely distributed Gram negative bacterium, considered one of the most important pathogens affecting salmonid farms worldwide producing a severe negative impact on this industry due to the high fish mortality rate caused by this pathogen and the costs associated with its chemical treatment (Nilsen et al. 2011). In adult salmonids the pathogen commonly produces extensive necrotic lesions, while in juveniles it produces an acute septicaemic infection (Cipriano and Holt 2005), but little is known about the pathogenesis of *F*. *psychrophilum*. The initial stage of infection is probably by adherence to the gills and intestine (Kondo et al. 2002; Bernardet and Bowman 2006), which is characteristic of virulent strains (Nematollahi et al. 2003). Adherence to surfaces is also the first stage in the formation of biofilms (Sauer et al. 2002). In aquaculture, biofilms are ubiquitous, appearing on the surfaces of water supply pipes, tanks, incubators, equipment components and bio-filtration systems, and even on the internal and external surfaces of fish, suspended matter, and different types of materials in fry and smolt cultivation tanks (King et al. 2004). Not only bacterial biofilms are frequently resistant to antibacterials and biocides, but they also have an important role as reservoirs of pathogens, enabling these to persist in aquaculture environments for a long period of time (Wingender and Flemming 2011). Furthermore, it is known that when *F*. *psychrophilum* forms biofilms at densities greater than 10^7^ cfu ml^-1^, it is less susceptible to antimicrobial agents, allowing cells which are resistant to sub-inhibitory concentrations of antibiotics to be selected rapidly (Sundell and Wiklund 2011).

The development of bacterial resistance to antibiotics has meant that there has been considerable interest in studying alternative treatments based on biocontrol, exploiting the antagonism against pathogens exercised by certain microorganisms (Maeda et al. 1997). Antagonistic interactions between species have been studied principally in the planktonic phase (Robertson et al. 2000; Kei et al. 2006; Ström-Bestor and Wiklund 2011), however, species of microbes included in biofilms can interact in various ways with other species within the same biofilm (Moons et al. 2009). These interactions start to influence a biofilm during the initial stages of its formation, adherence of the bacteria to the surface and colonization, and continue to influence the structure and physiology of the biofilm as it develops. The characteristics of biofilm growth suggest that the interactions between bacteria in biofilms is different from that occurring in the planktonic phase, making it impossible to predict which species will prevail in a biofilm system (Simões et al. 2008). It is believed that these interactions may be even more important in biofilms than in the planktonic state, because cell positions are relatively stable, and local areas of the biofilm which hinder molecular diffusion (James et al. 1995; Eberl and Collinson 2009).

Current studies on biofilm antagonism have searched for products extracted from bacteria which exhibit an anti-biofilm potential, for example, quorum-sensing inhibitors (Rasmussen and Givskov 2006; You et al. 2007; Defoirdt et al. 2011,) as well as other compounds which interfere with the formation of biofilms, such as iron chelators (Singh et al. 2002; Banin et al. 2005). In aquaculture, bacterial antagonism in planktonic state is the basis for the development of probiotics. These are already being used in fish and shrimp farming and can be administered in the feed or directly into the rearing tank in order to avoid infections with pathogenic bacteria, improving the growth and development of reared organisms (Sihag and Sharma 2012). At present however, no commercial probiotic treatment exists for the control of the fish pathogen *F*. *psychrophilum*. The genus *Carnobacterium*, which has been used as a probiotic for Atlantic salmon and Rainbow trout (Robertson et al. 2000), exhibited an inhibitory activity against *F*. *psychrophilum in vitro*, but failed to control this pathogen *in vivo*. Otherwise, strains belonging to the *Pseudomonas* (Korkea-Aho et al. 2011) and *Enterobacter* (Burbank et al. 2011) genus exhibited good results in *in vivo* tests, but have been not yet applied in mass cultures in order to evaluate their effectiveness at commercial scale. Unfortunately, these recent successful advances have been only addressed by using antagonists in the planktonic state, and it is not known the effect that antagonists may have on the formation of *F*. *psychrophilum* biofilm.

Although it is not considered necessary for all probiotics to form biofilms to be successful *in vivo*, interface colonisation is often a pre-requisite for probiotic effectiveness (Eberl et al. 2010). The disadvantage of probiotics is that they are not self-sustaining in the environment, needing to be re-applied regularly at high concentrations (Defoirdt et al. 2007), thus it is reasonable to consider the use of antagonist biofilms which persist for more time in the aquatic environments.

The main aim of this study was to determine the ability of an antagonistic biofilm to inhibit the biofilm formation of the fish pathogen *F*. *psychrophilum*. We present evidence that a biofilm of a *Pseudomonas fluorescens* strain is able to inhibit *F*. *psychrophilum* biofilm formation, mainly due to the production of siderophores.

## Results

### Identification of FF48 strain

FF48 strain was characterized as a Gram-negative rod exhibiting an oxidative metabolism of glucose and a fluorescent pigmentation, and the production of the catalase, oxidase and gelatinase enzymes. When a fragment of ARNr16S gene from the FF48 strain was sequenced and the alignment comparison of the sequence using BLAST (Basic Local Alignment Search Tool) (Altschul et al. 1990) was performed a 100% of identity and a score of 1157 with *Pseudomonas fluorescens* strain B15 (accession number AY581137) was found. The sequence was deposited in GenBank as *Pseudomonas fluorescens* with accession number KC602116.

### Inhibitory effect of supernatant of antagonist strain

**Figure 1:**
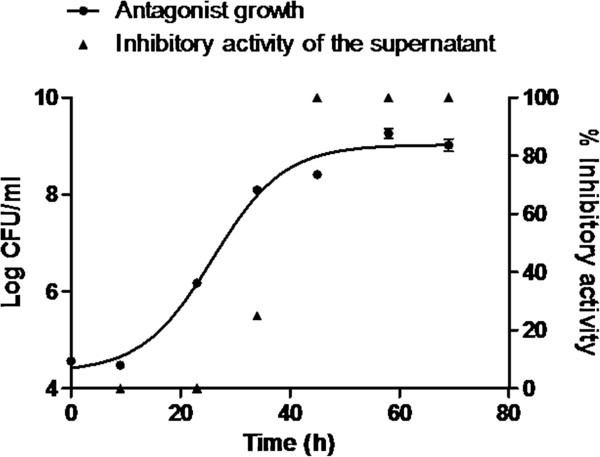
**Inhibitory activity of supernatant of*Pseudomonas fluorescens*FF48 on*F. psychrophilum*during the different growth phases of the antagonist.** It may be observed that inhibitory activity starts in the exponential growth phase of the FF48 strain and remains constant in the stationary phase. The graph includes the growth curve of the antagonist.

Maximum inhibitory dilution (MID) was obtained for each supernatant over time. To relate the MID values to the activity of the supernatant, it was assumed that the greater the inhibitory dilution of the supernatant, the greater would be its inhibitory activity on *F*. *psychrophilum* in a determined time. Relative quantification was done using the maximum inhibitory activity obtained in the assay as a reference. This was established as 100% of the inhibitory activity corresponding to the supernatant with the greatest MID. By evaluating the activity of the supernatant of *P*. *fluorescens* FF48 over 72 h, it was possible to determine the growth phase of the antagonist when the greatest inhibitory activity of its supernatant occurred. It was observed that the inhibitory activity of FF48 strain was only observed after 30 h of growth, increasing sharply entering stationary growth phase and remaining constant along the late-stationary phase, suggesting that the inhibitor compound is mainly produced during the stationary phase (Figure 1). When the greatest MID was determined, it was found that the supernatant maintained its inhibitory effect up to a dilution of 1/8. From this result a sub-inhibitory MID (1/16) was also determined, which was the concentration used subsequently in biofilm formation inhibition assays in the microplates. This concentration was used so that inhibition in the planktonic phase would not be considered as biofilm inhibition.

### Siderophore production

**Figure 2:**
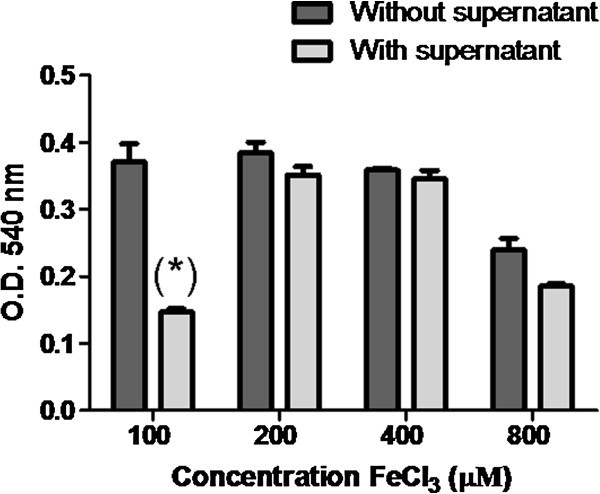
**Inhibition of*Flavobacterium psychrophilum*in the planktonic phase by supernatant of*Pseudomonas fluorescens*FF48 in the presence of different concentrations of iron (FeCl**_**3**_**) in half-strength NB medium.** The growth of *F*. *psychrophilum* with no supernatant is shown as a control. The growth of *F*. *psychrophilum* was determined from the optical density at 540 nm. The graph shows the average of the triplicates and the standard deviation.

To study the antagonist mechanism of *P*. *fluores*cens FF48 and its effect on *F*. *psychrophilum*, siderophore production by the antagonist strain was first explored using CAS agar assay. It was found that the FF48 strain produced a siderophore, evidenced by the loss of colour in CAS agar. According to the liquid CAS assay, it was determined that siderophores were also present in the supernatant, whereas by using spectrophotometric tests it appears that they probably belong to a hydroxamate siderophore, since, a peak was detected at 430 nm in the FeCl_3_ test. In order to study whether this siderophore detected in the supernatant had any effect on the growth of *F*. *psychrophilum*, the effect of the antagonist supernatant was tested under conditions of iron saturation, since, if the siderophore is responsible for the inhibition, loss of its activity should be detected under such conditions. It was found that the inhibitory effect, at a concentration of 200 µM of FeCl_3_ and upwards, was significantly lower than that observed at 100 µM (p<0.05) (Figure 2).

### Effect of supernatant of *Pseudomonas fluorescens* FF48 on the formation of *F*. *psychrophilum* biofilms

**Figure 3:**
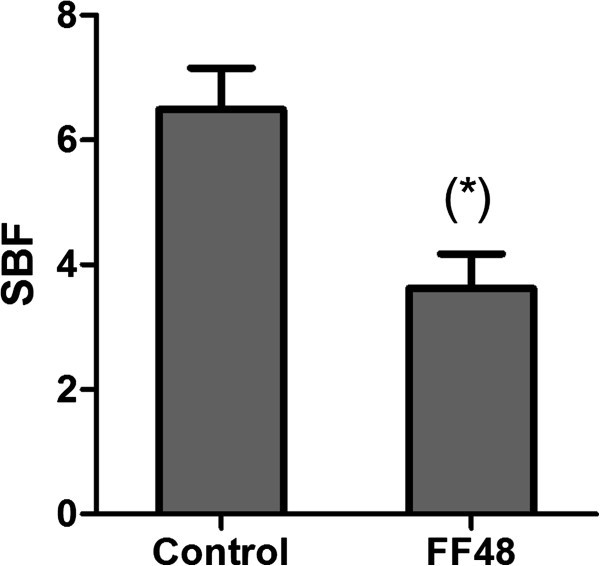
**Inhibition of*Flavobacterium psychrophilum*biofilm formation by supernatant of*Pseudomonas fluorescens*FF48.** The graph shows the SBF (Specific Biofilm Formation) index. Control without supernatant is included. The significant difference (p<0.05) against the control was determined by Anova and Tukey's post-test.

It was found that the supernatant of FF48 strain was able to significantly inhibit (p< 0.05) the formation of *F*. *psychrophilum* biofilms, since a lower SBF (Specific Biofilm Formation) index than the observed in the control without supernatant was obtained (Figure 3).

### Biofilm formation kinetics

**Figure 4:**
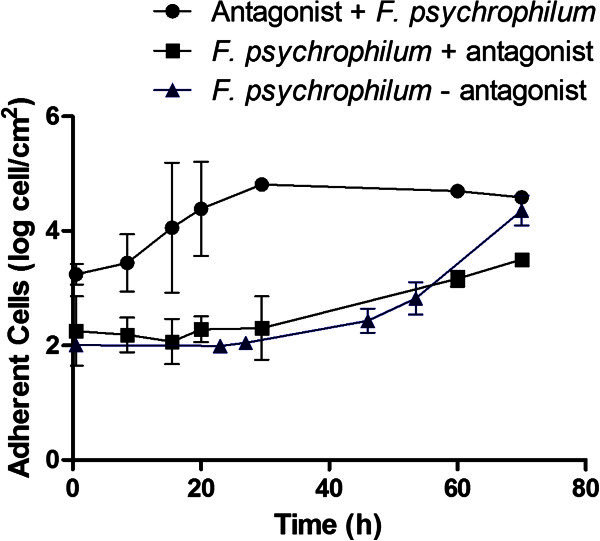
**Formation of*Flavobacterium psychrophilum*biofilm in presence of*Pseudomonas fluorescens*FF48.** Quantification of adhered *F*. *psychrophilum* cells in a dual-biofilm experiment corresponds to “*F*. *psychrophilum* + antagonist”, and adhered antagonist cells to the “Antagonist + *F*. *psychrophilum*”. The control, corresponding to the formation of *F*. *psychrophilum* biofilms in the absence of the antagonist (*F*. *psychrophilum* – antagonist), is also included. The values shown represent the average of 3 replications, together with the standard deviation.

**Figure 5:**
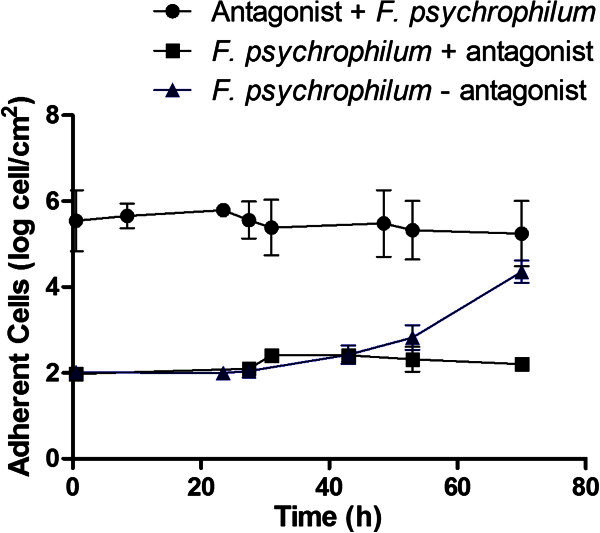
**Formation of*Flavobacterium psychrophilum*biofilm in the presence of a previously formed antagonist biofilm of*Pseudomonas fluorescens*.** Quantification of adhered *F*. *psychrophilum* cells corresponds to “*F*. *psychrophilum* + antagonist”. The quantification of adhered antagonist cells is also included; these appear as a stable biofilm throughout the experiment (*Antagonist control*). The control, corresponding to the formation of *F*. *psychrophilum* biofilms in the absence of the antagonist (*F*. *psychrophilum* – antagonist), is also included. The values shown represent the average of 3 replications, together with the standard deviation.

Once the antagonistic effect of the *P*. *fluorescens* FF48 on *F*. *psychrophilum* had been shown, the inhibition of the first stages of biofilm formation by the antagonist was studied. The results showed that when both biofilms were simultaneously formed, the antagonist biofilm was able to reduce the formation of *F*. *psychrophilum* biofilms on the support once the pathogen begins to adhere more rapidly, presenting a growth rate of 0.06730 ± 0.02 hˉ^1^, compared to 0.1902 ± 0.03 hˉ^1^ for *F*. *psychrophilum* alone on the support (Figure 4); however it did not completely inhibit the coexistence of the two species on the support. On the other hand, when the antagonist biofilm was formed first, it was able to inhibit totally the formation of *F*. *psychrophilum* biofilms up to 70 h after formation (Figure 5). It should be noted that the antagonist biofilm remained stable during the course of the experiment, at approximately 1×10^6^ cells per cm^2^.

## Discussion

The main purpose of this study was to determine the effect of the use of a biofilm formed by an antagonist strain on the formation of a fish pathogen biofilm. We evaluated the activity of the antagonist strain *P*. *fluorescens* FF48, previously selected by an agar screening test because its inhibitory activity on *F*. *psychrophilum*. On analysing the supernatant of FF48 strain, it was found that the supernatant exhibited an inhibitory activity even when was diluted 8 times, implying that this strain is able to release inhibitory compounds which may affect the growth of *F*. *psychrophilum* into the medium, agreeing with the results of Ström-Bestor and Wiklund (2011) for supernatant extracted from a strain of *Pseudomonas* sp.

Although it was not the main objective of this research, we were also interested in to detect siderophores in the supernatant of the antagonist to confirm its inhibitory activity because it is known that these molecules are frequently produced by strains belonging to the *Pseudomonas* genus, being responsible for the inhibition of other bacteria at low iron concentrations (Cornelis and Matthijs 2002; Visca et al. 2007; Cornelis 2010; Korkea-aho et al. 2011). In this work we found that FF48 strain produces a siderophore molecule preliminarily catalogued as a hydroxamate type, but it is necessary to evaluate whether these molecules could in fact be responsible for inhibiting *F*. *psychrophilum* because some reports indicated that *F*. *psychrophilum* species also produces siderophores, albeit of low affinity (Møller et al. 2005). To address this goal, an inhibition assay at iron saturation concentrations was developed, observing that the effect of the antagonist supernatant was lost from 200 µM of FeCl_3_, agreeing with the results of Ström-Bestor and Wiklund (2011), who observed a loss of inhibitory effect from 100 µM of FeCl_3_ to 400 µM. When a concentration of 800 µM of FeCl_3_ was applied, a reduction in the growth of *F*. *psychrophilum* was observed without the addition of supernatant of FF48 strain, indicating that at this concentration the excess of FeCl_3_ is toxic for bacterial growth. These results confirm the participation of an iron-dependent inhibition mechanism in the inhibitory activity of supernatant, which would probably be of the siderophore type, but further studies, such as mass spectrometry analysis must be performed to confirm the structure of this molecule. If this is the case, the affinity for iron of the FF48 siderophore would be much stronger than the affinity of the siderophores of *F*. *psychrophilum*, without discarding the possible direct inhibitory effect of the siderophore molecules, as was previously described (Gill and Warren 1988; Matthijs et al. 2007; Cornelis 2010). Furthermore, the detection and expression of genes encoding for the synthesis of siderophores as well as for siderophore receptors could clarify the functionality of the detected siderophore. Otherwise, the feasibility that other molecules, not related to the iron capture process could be responsible for the inhibitory activity cannot be discarded.

Subsequently we studied the interaction between the antagonist bacterium which exhibited inhibitory activity in its supernatant, and biofilm formation by *F*. *psychrophilum*. It was found that the supernatant of the antagonist at a sub-inhibitory concentration was able to affect the formation of *F*. *psychrophilum* biofilms, indicating that the inhibitory compound reduces biofilm formation even at a concentration which permits planktonic growth. The SBF index relates cells in biofilms with planktonic cells; it may therefore, be concluded that supernatant inhibit the growth of planktonic *F*. *psychrophilum* cells less than those in the biofilm. In this trend, we can hypothesise that sub-inhibitory concentrations of the inhibitory compound can capture the iron playing an important role in the biofilm formation process, without affecting the bacterial planktonic phase (Singh et al. 2002; Ishida et al. 2011). Otherwise, it cannot be discarded the possibility that another compound produced by the antagonist, not related to the iron capture process, could be responsible for the inhibition of the biofilm production (Defoirdt et al., 2011).

When a dual-biofilm was formed, the two bacteria start to adhere to a support despite the competitive advantage of the antagonist, observing that both bacteria were able to coexist on the support. This fact was similar to findings previously described in planktonic co-cultures (Gram et al. 1999; Vaseeharan and Ramasamy 2003) in which the antagonist bacteria is able to co-exist unless the antagonist is inoculated at a greater concentration than the pathogen. Something similar has also been observed in studies of mixed biofilms, with the two types of bacteria able to co-exist in a biofilm when they are inoculated simultaneously, since separate microcolonies are formed on the surface, with each species occupying a different space (Tait and Sutherland 2002; Kreth et al. 2008).

In our results it was observed that after the initial phase of co-existence, the *F*. *psychrophilum* biofilm started to form after 29 h, but more slowly (growth rate = 0.06730 ± 0.02 hˉ^1^). This phenomenon may be explained because initially the inhibitory compound was not present in the concentration necessary to be effective, as seen in Figure 1. It may be that during this time the formation of microcolonies of *F*. *psychrophilum* protects the bacteria from the action of an inhibitory compound. Later, as time passes, the *F*. *psychrophilum* biofilm starts to form, but more slowly, indicating that the inhibitory compounds are having an effect on the biofilm even when inhibition is not complete, suggesting that inhibitory activity on a biofilm in formation is different to the acting on a formed or mature biofilm. When the antagonist biofilm has been previously formed, a complete inhibition of the formation of the *F*. *psychrophilum* biofilm is observed. The explanation of this situation may be that the molecules only achieve their effect once the antagonist has already formed a biofilm. According to Kreth et al. (2008), if an antagonist biofilm is already formed, the time needed to produce the compound is reduced, being available in the medium when the pathogen arrives. Another factor to be considered is the type of mechanism involved, since although there is some evidence that siderophores play an important role in biofilm inhibition, it is conceivable that the participation of this mechanism is more important when the number of antagonist cells is high, then reducing the availability of iron, as occurs in the previously formed antagonist biofilm. Furthermore, we have preliminary results that support the hypothesis that the inhibition of biofilm formation is due to the iron deficiency. When the effect of *P*. *fluorescens* FF48 on the formation of a biofilm of *F*. *psychrophilum* in a CDC biofilm reactor, under different ferrous concentrations was evaluated, we found an inhibition of the colonization of *F*. *psychrophilum* only in absence of iron.

Finally, we would like to note that the use of real time PCR in this study enabled us to distinguish more exactly the two bacterial species, unlike the traditional methods of plate count and identification by microscopic morphology, still frequently used. We consider that in a plate count assay of the two species, an inhibition is generated on the plate which alters the results. In the case of morphological differentiation, identification may be subjective and in some cases difficult to perform, since the morphology of the bacteria may change when they form biofilms (Simões et al. 2008).

## Conclusions

In conclusion, the biofilm of *P*. *fluorescens* FF48 was effective in controlling the formation of *F*. *psychrophilum* biofilms *in vitro*. This is important, since the biofilm is the state in which microorganisms are highly persistant in the environment; therefore when a biocontroller is to be applied, the interaction between the antagonist-pathogen biofilms must be considered. As is shown in this work, the ideal situation is to ensure the prior establishment of the biocontroller in the cultivation system before it is confronted with the pathogen, to prevent the latter from adhering and persisting in the environment.

## Methods

### Bacterial strains and culture conditions

*Flavobacterium psychrophilum* strain 19749 (Company code), isolated from a moribund salmon displaying typical symptoms of the bacterial cold-water disease such as skin ulcers and fin lesions was purchased from the company Aquagestión S.A., Chile. It was cultured in half-strength nutrient broth (NB) (Difco™ Le Point de Claix, France) at 15°C. The identity of the species was confirmed by PCR using the primer pair PSY-1/ PSY-2 for *F* (Toyama et al. *psychrophilum*^1994^). The antagonist strain FF48 was isolated from the sediment under a Chilean freshwater fish farm (Miranda and Rojas 2007). This strain was selected because it showed inhibiting properties against *F*. *psychrophilum* in an antagonism screening agar diffusion test (data no shown), using the method described by Marja Tiirola, PhD., (University of Jyväskylä, Department of Biological and Environmental Science, Finland), and the antagonist strain, *Pseudomonas* sp. MT5 (Tiirola et al. 2002) was used as a positive control in the antagonism screening.

### Identification of the FF48 strain

Phenotypical characteristics, including Gram stain, oxidation/fermentation of glucose, fluorescent pigment, catalase, gelatinase and oxidase production were determined as described by Barrow and Feltham (1993). Molecular analysis was performed extracting genomic DNA of the antagonist strain using a commercial genomic DNA purification kit (Wizard, Promega, Madison, Wisconsin), and the 16S ribosomal RNA gene was amplified with primers 27F (5’- AGA GTTT GAT CCT GGT CAG AAC GCT-3’) / 1492R (5'-TAC GGC TAC CTT GTT ACG ACT TCA CCC C-3') (Jin et al. 2011). PCR amplification was conducted with AmpliTaq DNA Polymerase (Applied Biosystems, Foster City, California), with 25 cycles of 94°C for 1 minute, 56°C for 1 minute, and 72°C for 1 minute. The PCR products were analysed by electrophoresis in 1.5% agarose gels, and then purified and sequenced using the same primers at Macrogen Corp., USA.

### Evaluation of the inhibitory activity of supernatant of FF48 strain on *F*. *psychrophilum*

Supernatant was extracted from a culture of FF48 strain over time to examine the inhibitory activity of possible compounds released into the medium during growth. A culture of the FF48 strain in half-strength NB was started from an inoculum of 5×10^4^ cfu ml^-1^ determined by a plate count method and supernatants were extracted during growth of the culture every 12±2 h for a period of 72 h. The supernatants were obtained by removing every time 3 ml from culture, which were centrifuged at 10,000 rpm for 10 min followed by filtration through a 0.2 µm syringe filter (Millipore). In addition, samples were taken to determine the growth curve of the antagonist strain by using a spread plate count method. Serial two-fold dilutions of each antagonist supernatant from 1/2 to 1/32 were prepared in a flat-bottom 96-well microplate (Nunclon, Nunc, Myriad Industries, San Diego, CA, USA) to determine the maximum inhibitory dilution (MID), defined as the maximum dilution of supernatant which would inhibit bacterial growth. The serial dilutions were prepared in the wells initially by mixing 100 µl half-strength NB medium with 100 µl of supernatant. Each well series then received 10 µl of a 1/100 suspension of the *F*. *psychrophilum* culture adjusted to an OD_540nm_= 0.2 (approximately 3×10^8^ cfu ml^-1^), obtaining a final concentration in the well of approximately 1.5×10^5^ cfu ml^-1^. In addition, control wells containing *F*. *psychrophilum* suspensions without added supernatant were included. Plates were read at naked eye after a 48 h period of incubation and growth was considered positive when an increase in the turbidity of the culture was detected. As the MID is equivalent to the inhibitory activity of the supernatant (Nantitanon et al. 2007), the results were expressed as a percentage of the maximum inhibitory activity obtained, using the following formula:

where % IA is the Percentage of Inhibitory Activity, FMID is the dilution factor which corresponds to the Maximum Inhibitory Dilution for every sampling period (samples were taken at 12, 24, 36, 48, 60 and 72 h) and FMIDg is the dilution factor corresponding to the highest Maximum Inhibitory Dilution value recorded from all the samples taken during the assay.

### Detection of siderophores and inhibition of *F*. *psychrophilum*

The FF48 strain was grown in the iron-depleted, chemically defined minimal medium MM9 (Antonenka 2007) for 24 h at 28°C with shaking (120 rpm). This solution was autoclaved and supplemented with 10 ml of glucose 20% sterilized by filtration (0.22 µm). When liquid cultures of FF48 strain were grown they were assayed for the chrome azurol sulphonate (CAS) agar plate test according to Schwyn and Neilands (1987). Liquid cultures were centrifuged at 10,000 rpm for 15 min and filtered at 0.2 µm with a syringe filter (Millipore) to obtain an undiluted, cell-free supernatant, which was examined for the presence of siderophores by the CAS assay (Schwyn and Neilands 1987). The nature of the siderophores produced in the supernatant was determined preliminarily by the FeCl_3_ test, examining the maximum absorption of the iron-siderophore complex using a UV–vis spectrophotometer (TU-1810S Split Beam). One ml of supernatant was mixed with 1 ml of aqueous FeCl_3_ (2%), and a spectrogram was then done between 400 and 500 nm, using supernatant without FeCl_3_ as blank. The complex ferric hydroxamate siderophore should exhibit an absorbance peak between 420 and 450 nm, and the ferric catecholate shows an absorbance peak at 495 nm (Jalal and Helm 1991). Copper carboxylates was determined adding 1 ml of supernatant to 1 ml of 250 µM CuSO_4_ and 2 ml of acetate buffer (pH 4.0) and spectrogram was done between 190–280 nm, where carboxylates show an absorbance peak (Shenker et al. 1992).

To determine the effect on *F*. *psychrophilum* inhibition, saturation concentrations of FeCl_3_ (Ström-Bestor and Wiklund 2011) were used considering that siderophores should not have any effect on *F*. *psychrophilum* if the bacterium has sufficient iron available. The assays were done on a 96-well microplate (Nunc) and 10 µl of a FeCl_3_ aqueous solution were added to obtain final concentrations of 100, 200, 400 and 800 µM in each well containing 90 µl of half-strength NB medium, 100 µl of the antagonistic supernatant and 10 µl of a suspension of *F*. *psychrophilum*. The antagonistic supernatant was obtained from a stationary growth-phase culture grown in half-strengh NB as previously described (without dilution), whereas the inoculum of *F*. *psychrophilum* (10 µl) was obtained from a dilution 1/100 of a suspension adjusted to an OD_540nm_= 0.2 (approximately 3 × 10^8^ cfu ml^-1^), obtaining a concentration in the well of approximately 1.5 × 10^5^ cfu ml^-1^. The microplate was incubated for 3 days at 15°C and 120 rpm. The bacterial growth was quantified at the end of this period by determining the optical density of *F*. *psychrophilum* at 540 nm. This assay was done using three replications and the average and the standard deviation were estimated.

### Effect of the supernatant on biofilm formation

The Crystal Violet staining method modified from Álvarez et al. (2006) was developed. A 24-well microplate (Nunc) was used and 4 replications were done. One ml of supernatant of the antagonist strain at a sub-inhibitory dilution (1:16) was placed in each well containing 1 ml of 15% strength NB medium (with a low level of nutrients) and the well was inoculated with 100 µl of a dilution 1/100 of a suspension *F*. *psychrophilum* adjusted to an OD_540nm_= 0.2 (approximately 3×10^8^ cfu ml^-1^), obtaining a concentration in the well of approximately 1.5×10^5^ cfu ml^-1^. In addition, control wells containing inoculum, medium, supernatant of *F*. *psychrophilum* but no supernatant of the antagonistic strain (negative control), as well as other control wells containing culture medium, supernatant of *F*. *psychrophilum*, but without the inoculum of *F*. *psychrophilum* (abiotic control) were included and PBS was used to obtain identical volume conditions compared to the wells containing supernatants. The microplate was left to incubate for 4 days at 15°C and 120 rpm to allow biofilms to form. At the end of this period the planktonic culture was removed from the well and its optical density at 540 nm was measured. The wells were then washed three times with sterile distilled water prior to staining with crystal violet (Certistain7®, Merk) at 1% for 30 min. The colorant excess was eliminated by three successive washes with sterile distilled water. Finally, the microplates were dried for 10 min and the crystal violet was solubilised with 1.5 ml of ethanol for reading at 595 nm. Biofilm formation was quantified using the formula SBF = (AB-CW)/G (Niu and Gilbert 2004), where SBF is the Specific Biofilm Formation index, AB is the optical density at 595 nm of the stained cells adhering to the well, CW is the optical density at 595 nm of the stained control wells containing culture medium free of bacteria (abiotic control) and G is the optical density at 540 nm of the cells growing in the culture medium. SBF was determined for wells with antagonist supernatant and without antagonist supernatant (negative control). The average and standard deviation of three replications were determined.

### Installation of a continuous-flow chemostat system

A system consisting of a 250 ml Erlenmayer flask containing 120 ml of 15% strength NB, with 30 polystyrene rings of 5 cm^2^ each as support was installed. These rings were cut from a polystyrene tube (16 ml, 16×125mm, BD Falcon™, USA). The flask was connected to supply and exit pipes, both made of silicon (Masterflex®, Coleparmer, USA). A peristaltic pump (Masterflex® L/S model 7554–95) was used to regulate the entry and exit flow of the flask. The assembly was autoclaved for 20 min before inoculation, except for the rings which were sterilised with ethanol and then boiled in sterile water. The chemostat was kept in a cool room at 16°C, with shaking and aeration, throughout the experiment (70 h). A bacterial inoculum of 1×10^5^ cfu ml^-1^ was used for each bacterium in the flask. Before starting to change the medium, the system was kept at batch conditions for 4 h to allow cells to adhere to the support and the bacteria to become acclimatised. The flow was established at 1.5 ml min^-1^, on the basis of the criteria proposed by Komlos et al. (2005), sufficient to avoid the accumulation of suspended cells, according to the specific growth rate of each bacterium. These specific growth rates were 0.39 hˉ^1^ for *P*. *fluorescens* FF48 and 0.36 hˉ^1^*F*. *psychrophilum*, obtained from a growth curve determined by a plate count method under the same conditions of nutrient medium and temperature used in the assay of biofilm formation (72 h, 15% strength NB and 16°C). The dilution rate (medium flow rate/ culture volume) in the chemostat was 0.75 h^-1^, value higher than the growth rates of each bacteria. Maintaining the chemostat under the operating conditions described above, two inoculation conditions were assayed. Firstly, the two types of bacteria were inoculated simultaneously into the chemostat, while in the second assay the antagonist biofilm was allowed to form on the rings for 48 h before inoculation with *F*. *psychrophilum*. Independently, a *F*. *psychrophilum* biofilm was formed in the absence of an antagonist in a third chemostat as a control. Each experiment was replicated 3 times, and the average and standard deviation were estimated.

### Quantification of antagonist and pathogen under chemostat conditions

The system previously described was used and two rings were taken from each chemostat twice per day in aseptic conditions. They were washed in sterile water and then sonicated (Branson B1510 Ultrasonic Cleaner) in 2 ml of sterile water to re-suspend the cells. Cell DNA was extracted using the Wizard®Genomic DNA Purification kit (Promega, USA), and absolute quantification carried out using real time PCR with a Roche™ LightCycler 2.0. First, the standard curves for each bacterium were drawn from a PCR product as described by the manufacturer. The primers used for *F. psychrophilum* were those described by Toyama et al. (1994), whereas for *P*. *fluorescens* FF48 primers PS-FW (5´-AAGTTGGGAGGAAGG-3’) and PS-Rv (5’-ACACAGGAAATTCCACCACCC-3’) were used. The real time PCR was taken in 20 µl of reaction mixture containing 10 µl of Roche Master mix LightCycler (which already contains Sybergreen, dNTPS and Taq), 0.6 µl of each Primer, 0.2 µl of BSA, 6.6 µl of water and 2 µl of DNA template. The running programmes for *F*. *psychrophilum* were: (i) 95°C for 10 min (ii) 45 cycles of 95°C for 40 s, 60°C for 40 s, 72°C for 1 min; and for FF48: 40 cycles of 95°C for 60 min, 44°C for 60 min and 72°C for 60 min. All the samples were run in triplicate. The melting point was determined at 65°C for 10 min.

### Statistical analysis

The data were analysed using GraphPad Prism 5.0 statistical software. The slopes of the curves for biofilm formation in the chemostat were analysed using linear regression. The results, shown as mean ± S.D., were analysed using an Anova and Tukey’s post-test. A difference of *p*<0.05 was considered significant.

## Authors’ information

MD is PhD in Biological Sciences and currently responsible of biocontrol in a hatchery of molluscs in the Centro Regional de Estudios Ambientales, UCSC, Chile.

JMV is PhD student in Chemical Engineering Sciences and he works in biofilm control of pathogen of aquaculture industries, in the Centro de Biotecnología, UDEC, Chile.

CDM is PhD in Biological Sciences and is the Chief of the Aquatic Pathobiology Lab belonging to the Universidad Católica del Norte and he also belongs to the Centro de Estudios Avanzados en Zonas Aridas, Chile.

GG is PhD in Biological Sciences and is the Chief of the Antibiotics Lab belonging to the Universidad de Concepcion, Chile.

HU is PhD in Environmental Sciences and works in biofilms and environmental microbiology in Centro de Biotecnología, UDEC, Chile.

## Abbreviations

NB: Nutrient broth
MID: Maximum inhibitory dilution
CAS: Chrome azurol S
SBF: Specific biofilm formation
BSA: Bovine serum albumin
OD: Optical density.

## References

[CR1] Altschul SF, Gish W, Miller W (1990). Basic local alignment search tool. J Mol Biol.

[CR2] Álvarez B, Secades P, Prieto M (2006). A mutation in *Flavobacterium psychrophilum tlpB* inhibits gliding motility and induces biofilm formation. Appl Environ Microbiol.

[CR3] Antonenka U (2007). Factors and mechanisms of mobility of the high pathogenicity island of Yersinia.

[CR4] Banin E, Vasil ML, Greenberg EP (2005). Iron and *Pseudomonas aeruginosa* biofilm formation. PNAS.

[CR5] Barrow GI, Feltham RKA (1993). Cowan and Steel's Manual for the identification of medical bacteria.

[CR6] Bernardet JF, Bowman JP, Dworkin M, Falkow S, Rosenberg E, Schleifer KH, Stackebrandt E (2006). The Genus *Flavobacterium*. The Prokaryotes. A handbook on the biology of bacteria, vol 7.

[CR7] Burbank DR, Shah DH, LaPatra SE (2011). Enhanced resistance to coldwater disease following feeding of probiotic bacterial strains to rainbow trout (*Oncorhynchus mykiss*). Aquaculture.

[CR8] Cipriano RC, Holt RA (2005). *Flavobacterium psychrophilum*, cause of Bacterial Cold-Water Disease and Rainbow Trout Fry Syndrome. Fish disease leaflet 86.

[CR9] Cornelis P (2010). Iron uptake and metabolism in pseudomonads. Appl Microbiol Biotechnol.

[CR10] Cornelis P, Matthijs S (2002). Diversity of siderophore-mediated iron uptake system in fluorescent pseudomonads: not only pyoverdines. Environ Microbiol.

[CR11] Defoirdt T, Boon N, Sorgeloos P (2007). Alternatives to antibiotics to control bacterial infections: luminescent vibriosis in aquaculture as an example. Trends Biotechnol.

[CR12] Defoirdt T, Sorgeloos P, Bossier P (2011). Alternatives to antibiotics for the control of bacterial disease in aquaculture. Curr Opin Microbiol.

[CR13] Eberl HJ, Collinson S (2009). A modeling and simulation study of siderophore mediated antagonism in dual-species biofilms. Theor Biol Med Model.

[CR14] Eberl HJ, Khassehkhan H, Demaret L (2010). A mixed-culture model of a probiotic biofilm control system. Comput Math Methods Med.

[CR15] Gill PR, Warren GJ (1988). An iron-antagonized fungistatic agent that is not required for iron assimilation from a fluorescent rhizosphere pseudomonad. J Bacteriol.

[CR16] Gram L, Melchiorsen J, Spanggaard B (1999). Inhibition of *Vibrio anguillarum* by *Pseudomonas fluorescens* AH2, a possible probiotic treatment of fish. Appl Environ Microbiol.

[CR17] Ishida S, Arai M, Niikawa H (2011). Inhibitory effect of cyclic trihydroxamate siderophore, desferrioxamine E, on the biofilm formation of *Mycobacterium* species. Biol Pharm Bull.

[CR18] Jalal MA, Helm D, Winkelmann G (1991). Isolation and spectroscopic identification of fungal siderophores. Handbook of microbial iron chelates.

[CR19] James GA, Beaudette L, Costerton JW (1995). Interspecies bacterial interactions in biofilms. J Ind Microbiol.

[CR20] Jin F, Ding Y, Ding W (2011). Genetic diversity and phylogeny of antagonistic bacteria against *Phytophthora nicotianae* isolated from tobacco rhizosphere. Int J Mol Sci.

[CR21] Kei I, Yutaka N, Toshinao I (2006). Antagonistic activities of bacteria against the pathogen of cold-water disease, *Flavobacterium psychrophilum*. Umi no Kenkyu.

[CR22] King RK, Flick GJ, Pierson MD (2004). Identification of bacterial pathogens in biofilms of recirculating aquaculture systems. J Aquat Food Prod Technol.

[CR23] Komlos J, Cunningham AB, Camper AK (2005). Interaction of *Klebsiella oxytoca* and *Burkholderia cepacia* in dual-species batch cultures and biofilms as a function of growth rate and substrate concentration. Microb Ecol.

[CR24] Kondo M, Kawai K, Kurohara K (2002). Adherence of *Flavobacterium psychrophilum* on the body surface of the ayu *Plecoglossus altivelis*. Microbes Infect.

[CR25] Korkea-aho TL, Heikkinen J, Thompson KD (2011). *Pseudomonas* sp. M174 inhibits the fish pathogen *Flavobacterium psychrophilum*. J Appl Microbiol.

[CR26] Kreth J, Zhang Y, Herzberg M (2008). Streptococcal antagonism In oral biofilms: *Streptococcus sanguinis* and *Streptococcus gordonii* interference with *Streptococcus mutans*. J Bacteriol.

[CR27] Maeda M, Nogami K, Kanematsu M (1997). The concept of biological control methods in aquaculture. Hydrobiologia.

[CR28] Matthijs S, Tehrani KA, Laus G (2007). Thioquinolobactin, a *Pseudomonas* siderophore with antifungal and anti-Pythium activity. Environ Microbiol.

[CR29] Miranda CD, Rojas R (2007). Occurrence of florfenicol resistance in bacteria associated with two Chilean salmon farms with different history of antibacterial usage. Aquaculture.

[CR30] Møller JD, Ellis AE, Barnes AC (2005). Iron acquisition mechanisms of *Flavobacterium psychrophilum*. J Fish Dis.

[CR31] Moons P, Michiels CW, Aertsen A (2009). Bacterial interactions in biofilms. Crit Rev Microbiol.

[CR32] Nantitanon W, Chowwanapoonpohn S, Okonogi S (2007). Antioxidant and antimicrobial activities of *Hyptis suaveolens* Essential Oil. Sci Pharm.

[CR33] Nematollahi A, Decostere A, Pasmans F (2003). *Flavobacterium psychrophilum* infections in salmonid fish. J Fish Dis.

[CR34] Nilsen H, Olsen AB, Vaagnes Ø (2011). Systemic *Flavobacterium psychrophilum* infection in rainbow trout *Oncorhynchus mykiss* (Walbaum) farmed in fresh brackish water in Norway. J Fish Dis.

[CR35] Niu C, Gilbert ES (2004). Colorimetric method for identifying plant essential oil components that affect biofilm formation and structure. Appl Environ Microbiol.

[CR36] Rasmussen TB, Givskov M (2006). Quorum-sensing inhibitors as anti-pathogenic drugs. Int J Med Microbiol.

[CR37] Robertson PAW, O’Dowd C, Burrells C (2000). Use of *Carnobacterium* sp. as a probiotic for Atlantic salmon (*Salmo salar* L.) and rainbow trout (*Oncorhynchus mykiss*, Walbaum). Aquaculture.

[CR38] Sauer K, Camper AK, Ehrlich GD (2002). *Pseudomonas aeruginosa* displays multiple phenotypes during development as a biofilm. J Bacteriol.

[CR39] Schwyn B, Neilands JB (1987). Universal chemical assay for the detection and determination of siderophores. Anal Biochem.

[CR40] Shenker M, Oliver I, Helmann M (1992). Utilization by tomatoes of iron mediated by a siderophore produced by Rhizopus arrhizus. Journal of Plant Nutrition.

[CR41] Sihag RC, Sharma P (2012). Probiotics: the new ecofriendly alternative measures of disease control for sustainable aquaculture. J Fish Aquatic Sci.

[CR42] Simões M, Simões LC, Pereira MO (2008). Antagonism between *Bacillus cereus* and *Pseudomonas fluorescens* in planktonic systems and in biofilms. Biofouling.

[CR43] Singh PK, Parsek MR, Greenberg EP (2002). A component of innate immunity prevents bacterial biofilm development. Nature.

[CR44] Ström-Bestor M, Wiklund T (2011). Inhibitory activity of *Pseudomonas* sp. on *Flavobacterium psychrophilum*, *in vitro*. J Fish Dis.

[CR45] Sundell K, Wiklund T (2011). Effect of biofilm formation on antimicrobial tolerance of *Flavobacterium psychrophilum*. J Fish Dis.

[CR46] Tait K, Sutherland IW (2002). Antagonistic interactions amongst bacteriocin-producing enteric bacteria in dual species biofilms. J Appl Microbiol.

[CR47] Tiirola M, Valtonen ET, Rintamäki-Kinnunen P (2002). Diagnosis of flavobacteriosis by direct amplification of rRNA genes. Dis Aquat Org.

[CR48] Toyama T, Kita-Tsukamoto K, Wakabayashi H (1994). Identification of *Cytophaga psychrophila* by PCR targeted 16S ribosomal RNA. Fish Pathol.

[CR49] Vaseeharan B, Ramasamy P (2003). Control of pathogenic *Vibrio* spp. by *Bacillus subtilis* BT23, a possible probiotic treatment for black tiger shrimp *Penaeus monodon*. Lett Appl Microbiol.

[CR50] Visca P, Imperi F, Lamont IL (2007). Pyoverdine siderophores: from biogénesis to biosignificance. Trends Microbiol.

[CR51] Wingender J, Flemming HC (2011). Biofilms in drinking water and their role as reservoir for pathogens. Int J Hyg Environ Health.

[CR52] You J, Xue X, Cao L (2007). Inhibition of *Vibrio* biofilm formation by a marine actinomycete strain A66. Appl Microbiol Biotechnol.

